# Modeling of Soft Tissues Interacting with Fluid (Blood or Air) Using the Immersed Finite Element Method

**DOI:** 10.4236/jbise.2014.73018

**Published:** 2014-02

**Authors:** Lucy T. Zhang

**Affiliations:** JEC 2049, Department of Mechanical, Aerospace, and Nuclear Engineering, Troy, USA

**Keywords:** Biomechanics, Fluid-Structure Interactions, Biomechanics

## Abstract

This paper presents some biomedical applications that involve fluid-structure interactions which are simulated using the Immersed Finite Element Method (IFEM). Here, we first review the original and enhanced IFEM methods that are suitable to model incompressible or compressible fluid that can have densities that are significantly lower than the solid, such as air. Then, three biomedical applications are studied using the IFEM. Each of the applications may require a specific set of IFEM formulation for its respective numerical stability and accuracy due to the disparities between the fluid and the solid. We show that these biomedical applications require a fully-coupled and stable numerical technique in order to produce meaningful results.

## 1. INTRODUCTION

In the past decade, the interest in developing novel simulation techniques for modeling fluid-structure interactions revived due to the increasing demands in capabilities to accurately and efficiently study biomedical applications. Biomedical applications often involve fluid (blood) interacting with soft tissues. In some cases, the fluid can also be air, which has a disparate density compared to the soft tissues.

Since soft tissues come in with all forms, shapes and sizes, it is more convenient to set up a simulation using non-boundary-fitted modeling technique. The non-boundary-fitted approaches avoid the re-meshing process by defining independent meshes for the fluid and solid respectively. The solid can freely move on top of the fluid grid without deforming the surrounding fluid. A widely used numerical approach for bio-interface applications is the immersed boundary (IB) method, which was initially proposed by Peskin to study the blood flow around heart valves [1–7]. The mathematical formulation of the IB method employs a mixture of Eulerian and Lagrangian descriptions for fluid and solid domains. In particular, the entire fluid domain is represented by a uniform background grid, which can be solved by finite difference method with periodic boundary conditions; whereas the submerged structure is represented by a fiber or boundary network. The interaction between the fluid and structure is accomplished by distributing the nodal forces and interpolating the velocities between Eulerian and Lagrangian domains through a smoothed approximation of the Dirac delta function. The advantage of the IB method is that the fluid-structure interface is tracked automatically by following the displaced structural boundary movement, which removes the costly computations due to various mesh update algorithms. Many other numerical algorithms have been developed that are inspired by the IB method, such as the immersed interface method (IIM) [8–14], the extended immersed boundary method (EIBM) [15] and the immersed boundary finite element method (IB-FEM) [16,17]. A review on several methods can be found in [18].

The problem existing in the non-boundary-fitted approaches mentioned above is the lack of more realistic representations of the solid, which hinders the accurate assessment of the material behavior and its deformation. Since the solid and the fluid domains are fully-coupled, a slight inaccuracy in estimating the solid solution may even affect the surrounding fluid solutions. This problem may propagate over time and cause instabilities in the solution or convergence issues. The immersed finite element method (IFEM) [19–22] was developed to tackle this problem by representing the background viscous fluid with an unstructured finite element mesh and non-linear finite elements for the immersed deformable solid. Similar to the immersed boundary method, the fluid domain is defined on a fixed Eulerian grid. However, the solid domain is constructed independently with a Lagrangian mesh, which makes it possible to use a more detailed constitutive model to describe the solid material such as linear elastic, hyperelastic and viscoelastic. This approach is particularly attractive to modeling biomedical applications including stent deployment, blood flow in athersclerosis in arteries, etc. [23–31]. With finite element formulations for both fluid and solid domains, the submerged structure is solved more realistically and accurately in comparison with the corresponding fiber network representation in the IB method. The caveat, of course, is to remove the artificial fluid where the solid volume occupies. Since the solid moves at every time step, the artificial fluid also moves. Using the non-boundary-fitted approach, this volume can be easily identified. The fluid solver is based on a stabilized equal-order finite element formulation applicable to problems involving moving boundaries [32–34]. This stabilized formulation prevents numerical oscillations without introducing excessive numerical dissipations. It is also possible to assign sufficiently refined fluid mesh in local regions wherever necessary to obtain more accurate interfacial solutions.

The two-way coupled approach, *i.e*. the interpolation and the distribution of the velocity and the forces between the two domains, is quite robust when the solid behaves very much like the fluid. However, if there exists high discontinuity in density as well as other intrinsic parameters in the solid and the fluid, the force and the velocity to be interpolated between the two fields can no longer provide consistent convergence. Therefore, a semi-implicit algorithm for the immersed finite element [35] was developed to alleviate the situations when large density difference and/or stiff solid material are used in the solid domain. The calculation of the fluid-structure interaction force is modified in order to achieve a larger stabilization range.

The semi-implicit IFEM algorithm works well when the fluid dominates the dynamics of the system, in which the solid moves and deforms by following the fluid flow. However, when the solid dynamics or inertia must be taken into account, letting the solid follow the fluid movement may lead to unrealistic solid deformation and sometimes even causes the distortion of the solid mesh, because it is not appropriate to still approximate the solid behaviors using only the fluid velocity. A modified IFEM algorithm (mIFEM) [36] was then introduced to provide a more accurate prediction of the solid motion and deformation, which directly depend on the solid inertia effects, constitutive laws, and the fluid solutions near the fluid-structure interface. The results show that it produces more accurate and reasonable solid responses compared to the original IFEM algorithm.

In this paper, we first provide a detailed derivation and descriptions of the mathematical formulations for IFEM, the semi-implicit IFEM, and the modified IFEM. Then, we will show several biomedical applications that used IFEM algorithms. The choice of the algorithm used for each application is dependent on the nature of the fluid-structure interactions involved, which comes clear as the readers go through the motivations of each of the algorithm development.

## 2. KINEMATICS AND ASSUMPTIONS

### 2.1. Kinematics

**Figure 1:**
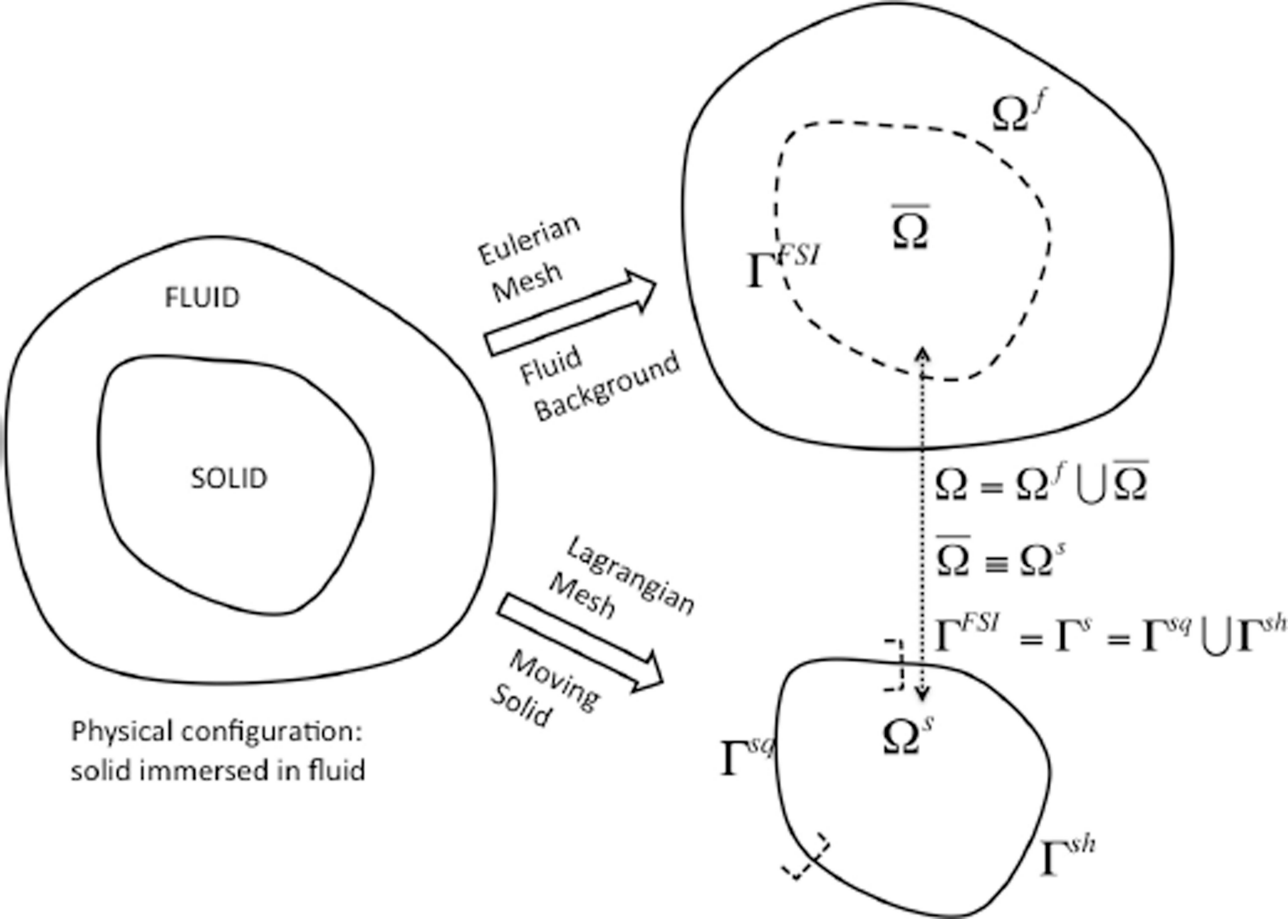
Computational domain decomposition.

Let us consider a deformable structure that occupies a finite domain, Ω^*s*^, which is completely immersed in a fluid domain Ω^*f*^, as illustrated in Figure 1. The fluid and the solid together occupy the entire computational domain Ω, and they intersect at a common interface Γ^*FSI*^, where “*FSI*” represents a line if Ω is a two-dimensional domain or a surface if Ω is in three-dimensions. The interface Γ^*FSI*^ coincides with the solid boundary Γ^*s*^. The nomenclature involved can be partitioned into two categories: one belongs to the solid and the other to the fluid. The notations associated with the solid have superscript *s* to distinguish them from those of the fluid *f*.

### 2.2. Assumptions

Before showing derivations, we first need to state three assumptions:

1. The fluid exists everywhere in the domain, Ω. This assumption allows us to generate fluid and solid meshes and solve fluid and solid equations independently, thus avoiding frequent mesh updating schemes required to track the fluid-structure interface. In the IFEM, the solid immersed in the fluid domain occupies a physical space or volume in the computational domain. Therefore, when the solid domain, Ω^*s*^, is constructed, it overlaps with the entire domain Ω filled with fluid. Since both the solid and the “artificial” fluid co-exist in Ω^*s*^, it is also referred to as the “overlapping domain”, Ω̅, *i.e*., Ω^*s*^ = Ω̅, in later text. This is illustrated in Figure 1. This assumption may simplify the computations, but does not comply with the actual physics. Therefore, this “artificial” fluid effect in the solid domain must be eliminated when formulating the equations.
2. The interface between the fluid and the solid must abide by the matching velocity (or no-slip) and traction boundary conditions. This assumption states that the sol id boundary moves together with the artificial fluid boundary or vice-versa, and the surface traction on both domains are equal and opposite. This assumption allows appropriate coupling to occur between the two domains.
3. The solid must always remain immersed in the fluid domain to avoid inaccurate interpolations at the fluid-solid interface.

### 2.3. Interpolations between Fluid and Solid Domains

The solid and fluid meshes are constructed independently, therefore it is impossible to have the moving solid boundary nodes exactly coinciding with the fluid nodes in Ω. An interpolation function, ϕ, must be used to couple the fluid velocity field ***v**^f^* (***x**^f^*, *t*) and the solid nodal velocity ***v**^s^* (***X**^s^*, *t*), such that:

(1) $$v_{i}^{s}(X^{s},t)=\int_{\Omega^{s}}v_{i}^{f}(x^{f},t)\phi (x^{f}-x^{s}(X^{s},t))d\Omega .$$

Similarly, the interaction force calculated in the solid (overlapping) domain ***f**^FSI,s^* (***X**^s^,t*) is distributed to the fluid domain ***f**^FSI,f^* (***x**^f^,t*) as:

(2) $$f_{i}^{FSI,f}(x^{f},t)=\int_{\Omega }f_{i}^{FSI,s}(X^{s},t)\phi (x^{f}-x^{s}(X^{s},t))d\Omega .$$

This two-way coupling is necessary to ensure the stability and convergence of the algorithm. There are multiple ways of performing the interpolation process. The interpolation function, ϕ, can be acquired through the discretized Dirac delta function [37], the sharp finite element interpolation function [22], or the reproducing kernel interpolation function [20]. The details and the characteristics of each approach can be found and compared in Ref. [22].

## 3. THE IMMERSED FINITE ELEMENT METHOD

The derivation of IFEM starts from the principle of virtual work or the weak form, which is used for standard finite element analysis. The weak forms of the derived equations are equivalent to their strong forms if the weak form solution is smooth enough to satisfy at least *C*^0^ continuity.

### 3.1. Derivation

The virtue work in the solid domain with a test function, **δ*v^s^*** can be expressed as:

(3) $$\int_{\Omega^{s}}\delta v_{i}^{s}[\rho^{s}\frac{dv_{i}^{s}}{dt}-\sigma_{ij,j}^{s}-\rho^{s}g_{i}]d\Omega =0.$$

The terms in the bracket of Equation (3) describe the governing equation for the solid, where **σ**^*s*^ is the stress which is directly related to the internal force that is determined by the material types and properties. The term $\rho^{s}(dv_{i}^{s}/dt)$, or ρ^*s*^*ü^s^*, is the inertial force and ρ^*s*^ *g_i_* is the body or external force.

To include the artificial fluid related terms without contradicting the equilibrium, Equation (3) can be rewritten as:

(4) $$\int_{\Omega^{s}}\delta v_{i}^{s}[(\rho^{s}-\underline{\rho^{f})\frac{dv_{i}^{s}}{dt}}+\underline{\rho^{f}\frac{dv_{i}^{s}}{dt}}-(\sigma_{ij,j}^{s}-\underline{\sigma_{ij,j}^{f})}\;-\underline{\sigma_{ij,j}^{f}}-(\rho^{s}-\underline{\rho^{f})g_{i}}-\underline{\rho^{f}g_{i}}]d\Omega =0.$$

The added terms are underlined. One can notice that they sum to zero. Since Ω^*s*^ and Ω̅ belong to the same physical space, we can rearrange this equation to yield:

(5) $$\int_{\Omega^{s}}\delta v_{i}^{s}[(\rho^{s}-\rho^{f})\frac{dv_{i}^{s}}{dt}-(\sigma_{ij,j}^{s}-\sigma_{ij,j}^{f})-(\rho^{s}-p^{f})g_{i}]d\Omega \;+\int_{\bar{\Omega }}\delta v_{i}^{s}(\rho^{f}\frac{dv_{i}^{s}}{dt}-\sigma_{ij,j}^{f}-\rho^{f}g_{i})d\Omega =0.$$

Equation (5) now contains two integral terms. The first term is the work done by the solid in the solid domain subtracting the work done by the artificial fluid. The second term represents the work done by the artificial fluid in this overlapping domain.

We define the first term in Equation (5) to be the interaction force:

(6) $$-f_{i}^{FSI,s}=(\rho^{s}-\rho^{f})\frac{dv_{i}^{s}}{dt}-(\sigma_{ij,j}^{s}-\sigma_{ij,j}^{f})-(\rho^{s}-\rho^{f})g_{i}.$$

Since this interaction force is first evaluated in the solid domain on the solid nodal points, it is therefore labeled as *f^FSI,s^*. It represents the interaction force acting on the solid from the fluid. Once it is evaluated, the nodal forces are then distributed onto the fluid domain as *f^FSI,f^*. This force, then, becomes the driving force for the fluid. Equation (5) becomes:

(7) $$\int_{\bar{\Omega }}\delta v_{i}^{s}(\rho^{f}\frac{dv_{i}^{s}}{dt}-\sigma_{ij,j}^{f}-\rho^{f}g_{i})d\Omega =\int_{\bar{\Omega }}\delta v_{i}^{s}f_{i}^{FSI,f}d\Omega .$$

Using the no-slip assumption made in Assumption (2) which allows $v_{i}^{f}=v_{i}^{s}$ in Ω̅, Equation (7) becomes:

(8) $$\int_{\bar{\Omega }}\delta v_{i}^{f}(\rho^{f}\frac{dv_{i}^{f}}{dt}-\sigma_{ij,j}^{f}-\rho^{f}g_{i}-f_{i}^{FSI,f})d\Omega =0.$$

Inside the bracket of this equation is the momentum equation for the artificial fluid, where all the terms resembles the Navier-Stokes equation except the addition of the interaction force *f^FSI,f^*. The interaction force only exists in the overlapping region and its immediate surroundings. Its value diminishes to zero at places outside the region.

Now, combining the work done by the real fluid and the artificial fluid described in Equation (8) with the expansion of the total time derivative term, $dv_{i}^{f}/dt=v_{i,t}^{f}+v_{j}^{f}v_{i,j}^{f}$, we obtain

(9) $$\int_{\bar{\Omega }}\delta v_{i}^{f}[\rho^{f}(v_{i,t}^{f}+v_{j}^{f}v_{i,j}^{f})-\sigma_{ij,j}^{f}-\rho^{f}g_{i}-f_{i}^{FSI,f}]d\Omega \;+\int_{\Omega^{f}}\delta v_{i}^{f}[\rho^{f}(v_{i,t}^{f}+v_{j}^{f}v_{i,j}^{f})-\sigma_{ij,j}^{f}-\rho^{f}g_{i}]d\Omega =0.$$

The two integral terms in Equation (9) can be combined into the entire computational domain, Ω, as:

(10) $$\int_{\Omega }\delta v_{i}^{f}[\rho^{f}(v_{i,t}^{f}+v_{j}^{f}v_{i,j}^{f})-\sigma_{ij,j}^{f}-\rho^{f}g_{i}-f_{i}^{FSI,f}]d\Omega =0.$$

Since the fluid is homogenous and both physical and artificial fluids are assumed to be incompressible, we can write the complete governing equations of fluid as

(11) $$v_{i,i}^{f}=0,$$

(12) $$\rho^{f}(v_{i,t}^{f}+v_{j}^{f}v_{i,j}^{f})-\sigma_{ij,j}^{f}-\rho^{f}g_{i}-f_{i}^{FSI,f}=0.$$

In the IFEM formulation, the term $f_{i}^{FSI,f}$ can be interpreted as the external force applied to the fluid that is generated from the artificial fluid. It is important to note that since the solid nodal velocities follow that of the overlapping fluid grid velocities, the compressibility of the solid must follow that of the fluid as well. Therefore, the solid must be incompressible or at least nearly incompressible when the fluid is incompressible. This restriction is alleviated in the modified IFEM algorithm in Section 5.

### 3.2. Outline of the IFEM Algorithm

An outline of the IFEM algorithm can be illustrated as follows:

1. Given the structural configuration ***x**^s^* and the fluid velocity ***v**^f^* from the previous time step *n* − 1,
2. Evaluate the nodal interaction forces ***f**^FSI,s^* on solid material points, using Equation (6),
3. Distribute the material nodal force onto the fluid grid, from ***f**^FSI,s^* to ***f**^FSI,f^* using interpolation function Equation (2),
4. Solve for fluid velocities ***v**^f^* and pressure *p^f^* implicitly using Equations (11) and (12) at current time step *n*,
5. Interpolate the velocities in the fluid domain to the material points, *i.e*. from ***v**^f^* to ***v**^s^*, as in Equation (1),
6. Update the positions of the structure using ***u**^s^* = ***v**^s^*Δ*t* and go back to step (1).

## 4. SEMI-IMPLICIT IFEM

In the IFEM, small time step has to be used to ensure the stability of the coupling procedure because the solid domain and fluid domain are coupled to each other explicitly at every time step. Since the Navier-Stokes equations are solved implicitly, such small time step requirement due to the coupling stability makes the whole algorithm numerically inefficient especially for the cases when the solid properties are very different from the fluid. Semi-implicit coupling between the fluid and solid domain is then introduced in order to enlarge the stability range.

### 4.1. Explicit Fluid-Structure Interaction Force

Although the force or the work is balanced seamlessly in the strong and weak forms at every time step, the fluid domain is numerically balanced with the fluid-structure interaction force evaluated based on the solid configuration of the *previous* time step. Therefore, the coupling between the two domains is considered explicit. In Equation (6) both the acceleration term (***ü**^s^*) and the solid internal stress term ∇·**σ**^*s*^ are evaluated based on the solid nodal velocity which is interpolated from the fluid velocity of the previous time step, *n* − 1:

(13) $$f^{FSI,s}=-{\left[(\rho^{s}-\rho^{f}){ü}^{s}\right]}^{n-1}+{\left(\nabla \cdot \sigma^{s}-\nabla \cdot \sigma^{f}\right)}^{n-1}\;+(\rho^{s}-\rho^{f})g\text{in}\Omega^{s}.$$

with two of the terms evaluated from time step *n* − 1, the interaction force is effectively (***f**^FSI,s^*)^*n*−1^. It is then passed onto the Navier-Stokes momentum equation to solve for ***v*** and *p* at the current time step *n* :

(14) $${\left(v_{,t}^{f}\right)}^{n}+{\left(v^{f}\right)}^{n}\cdot {\left(\nabla v^{f}\right)}^{n}+\frac{1}{\rho^{f}}{\left(\nabla p^{f}\right)}^{n}-\nu {\left(\nabla^{2}v^{f}\right)}^{n}\;-\frac{1}{\rho^{f}}{\left(f^{FSI,f}\right)}^{n-1}=0\text{in}\Omega .$$

Noting that every term in Equation (14) is solved in the current time step *n* except the last term where the interaction force is evaluated from the previous time step *n* − 1. The last term can be related to the current interaction force by taking the Taylor's expansion at time step *n*:

(15) $${\left(f^{FSI,f}\right)}^{n-1}={\left(f^{FSI,f}\right)}^{n}-{\left(f_{,t}^{FSI,f}\right)}^{n}\Delta t+O(\Delta t^{2}).$$

The error due to the explicit coupling can be approximated by substituting Equation (15) into Equation (14):

(16) $${\text{Error}}^{\text{Coupling}}=\frac{1}{\rho^{f}}f_{,t}^{FSI,f}(t)\Delta t+O(\Delta t^{2}).$$

Based on the definition of the fluid-structure interaction force in Equation (13), each term of this fluid-structure interaction force contributes to the accumulative coupling error. These terms are proportional to the density ratio ρ^*s*^/ρ^*f*^ − 1, the stiffness ratio *K*/ρ^*f*^ and the gravity ratio (ρ^*s*^ ρ^*f*^ − 1) *g*, respectively. Here, *K* is an equivalent Young’s modulus of the solid representing the stiffness of the solid material. If any of these terms are large, the resulting error due to the coupling would be large. These large errors often result in instability or divergence of the solution.

### 4.2. Semi-Implicit Fluid-Structure Interaction Force

To alleviate the numerical issues caused by the restrictions in time step size of explicit coupling and the convergence problem due to highly disparate properties between the fluid and the solid domains, a semi-implicit approach is introduced [35]. In the semi-implicit algorithm, the interaction force ***f**^FSI,s^* is re-defined in the solid domain, which *only* includes the internal forces for the fluid and solid from the original definition in Equation (13), such that:

(17) $$f^{FSI,s}=\nabla \cdot \sigma^{s}-\nabla \cdot \sigma^{f}\text{in}\Omega^{s},$$

The rest of the terms in the original explicit formulation Equation (13), namely, the inertial and external force terms, are now incorporated into the fluid equations. The newly defined **f**^*FSI,s*^ is distributed to the fluid domain as in the original IFEM. The Navier-Stokes equations now must also be re-defined as follows:

(18) $$\nabla \cdot v^{f}=0,$$

(19) $$\bar{\rho }(v_{,t}^{f}+v^{f}\cdot \nabla v^{f})=-\nabla p^{f}+\mu \nabla^{2}v^{f}+f^{FSI,f}+\bar{\rho }g\text{in}\Omega ,$$

where ρ̅ is defined as:

(20) $$\bar{\rho }=\rho^{f}+(\rho^{s}-\rho^{f})I(x).$$

Here, the indicator function, *I* (***x***), is to identify the real fluid region Ω^*f*^, the artificial fluid region or the solid region Ω^*s*^, and the fluid-structure interface Γ^*FSI*^, in the computational domain Ω. The value of the indicator function is ranged between 0 and 1 where it is 0 if an entire element belongs to the fluid and 1 if an entire element belongs to the solid. This newly revised fluid’s momentum equation combines the inertial and the gravity terms in the original FSI force equation.

To further improve the algorithm, we enhance the indicator function so that it can accommodate high density ratios between the fluid and the solid. This means that the interfacial elements have the transitional indicator values. To do so, the elements that contain the fluid-solid interface have varying indicator value that transits from 0 to 1 by solving Poisson’s equation [38]:

(21) $$\nabla^{2}I=\nabla \cdot G^{f},$$

where ***G**^f^* is interpolated by

(22) $$G^{f}=\int_{\Gamma^{s}}n\phi (x-x^{s})d\Gamma^{s}.$$

Here, ***n*** is the unit outward normal of the solid interface and ϕ (***x*** − ***x**^s^*) is the same interpolation function used by the velocity interpolation function and force distribution. The boundary conditions are given as,

(23a) $$I(x)=0,\text{in}\Omega^{f}$$

(23b) $$I(x)=1,\text{in}\Omega^{s}.$$

Since the fluid-solid interface moves, this indicator function is updated at every time step based on the relative position of the solid domain in the entire computational domain. Comparing to the original IFEM algorithm, the inertial and the external force terms in the original interaction force formulation are now been considered in the governing equation and can be evaluated iteratively with the most updated velocity field. This is, therefore, considered as semi-implicit IFEM algorithm.

In this formulation, the solid internal stress is still dependent on the fluid solutions from the previous time step. Even though this term is evaluated explicitly, the semi-implicit scheme still significantly improves the convergence of the solution when the solid material is very stiff. If we re-visit the coupling error Equation (16), the magnitude of the coupling error in the internal stress term is proportional to the stiffness ratio *K*/ρ̅. Noting that in the semi-implicit form, ρ̅ is defined in Equation (20); the stiffness ratio here is in fact *K*/ρ^*s*^. For most of the cases, the solid density will be larger than the fluid density, which reduces the coupling error comparing to *K*/ρ^*f*^ from the original explicit form. Therefore, although the solid internal force is still computed explicitly, the coupling error for the semi-implicit scheme is smaller than the explicit scheme.

Overall, this semi-implicit scheme relaxes the small time step requirement and ensures the stability of the FSI force estimation. In particular, this algorithm can handle a much larger range of fluid and solid properties without sacrificing the computational time.

### 4.3. Outline of the Semi-Implicit IFEM Algorithm

An outline of the semi-implicit IFEM algorithm is illustrated as follows:

1. Given the structural configuration ***x**^s^* and the fluid velocity ***v**^f^* from time step *n* − 1,
2. Evaluate the nodal semi-implicit interaction forces ***f**^FSI,s^* on the solid material points, using Equation (17),
3. Distribute the material nodal force onto the fluid grid, from ***f**^FSI,s^* to ***f**^FSI,f^* using delta function Equation (2),
4. Obtain the indicator field by solving Equation (21),
5. Solve for fluid velocities ***v**^f^* and pressure *p^f^* implicitly using Equations (18), (19) and (20),
6. Interpolate the velocities in the fluid domain to the material points, i.e. from ***v**^f^* to ***v**^s^*, as in Equation (1),
7. Update the positions of the solid nodes using ***u**^s^* = ***v**^s^*Δ*t* and go back to step (1).

## 5. THE MODIFIED IFEM

Although the semi-implicit coupling scheme alleviates some convergence issues when the fluid and solid have large property differences, one can notice that the solid *dynamics* is still been controlled by the artificial fluid. For cases where the solid behavior dominates the entire system or high Reynolds number flows using the IFEM algorithm may lead to unrealistic solid deformation and may even cause the severe distortion of the solid mesh, because it is not appropriate to approximate the solid behavior based on the fluid velocity by letting ***v**^s^* = ***v**^f^*. The idea of the modified IFEM is to let the artificial fluid to behave more like the solid, or letting ***v**^f^* = ***v**^s^*. Doing so allows the solid governing equation to be *solved* rather than be *evaluated*. Since the artificial fluid is not real anyway, its role is to produce the same velocity as the solid so that the real fluid realizes the existence of the solid. This modified IFEM algorithm allows the solid behaviors to be estimated more accurately and have stronger influences in the fluid-structure interactions. The detailed rationale and derivations, as well as validation cases were presented in [36].

### 5.1. Derivation

In order to find the solid displacement field ***u**^s^* and the velocity field ***v**^s^*, the solid equation is solved,

(24) $$\rho^{s}u_{i,tt}^{s}=\sigma_{ij,j}^{s}\text{in}\Omega^{s}.$$

The solid stress **σ**^*s*^ is evaluated using the solid strain tensor **ε**^*s*^,

(25) $$\sigma_{kl}^{s}=c_{ijkl}\varepsilon_{ij}^{s}+\eta_{ijkl}\varepsilon_{ij,t}^{s},$$

where $\varepsilon_{ij}^{s}=1/2(u_{i,j}^{s}+u_{j,i}^{s})$. Different combinations of *c_ijkl_* and η_*ijkl*_ provide various choices of solid material constitutive laws such as linear elastic, viscolinear elastic, hyper-elastic, etc.

The boundary condition of the solid domain can be applied using either Dirichlet boundary condition described in Equation (26) or Neumann boundary condition described in Equation (27).

(26) $$u_{i}^{s}=q_{i}=v_{i}^{f}\Delta t\text{on}\Gamma^{\text{sq}}.$$

(27) $$\sigma_{ij}^{s}n_{j}=h_{i}=-\sigma_{ij}^{f}n_{j}\text{on}\Gamma^{\text{sh}}.$$

Here, ***n*** is the outward normal of the fluid-structure interface Γ^*FSI*^. These boundary conditions are evaluated based on the fluid velocity (***v**^f^*) and stress (**σ**^*f*^) on the fluid-structure interface solved from the fluid equations at previous time step. Δ*t* is the time step size.

Once the solid solution is obtained, the next step is to make the artificial fluid to follow the solid, *i.e*. solving the artificial fluid governing equation so that *v^f^* = *v^s^* in Ω̅. To accomplish this, the artificial fluid property, such as the density, should mimic that of the solid.

The continuity equation of the artificial fluid (Ω̅) can be written as,

(28) $$\frac{\partial \rho^{s}}{\partial t}+{\left(\rho^{s}v_{i}^{f}\right)}_{,i}=0\text{in}\bar{\Omega }.$$

It is also necessary that the artificial fluid has the same compressibility (κ^*s*^) as the solid. Therefore, the artificial fluid is described as a pseudo-compressible fluid:

(29) $$\frac{1}{\rho^{s}}\frac{\partial \rho^{s}}{\partial t}=\frac{1}{\kappa^{s}}\frac{\partial p^{f}}{\partial t},$$

where the compressibility of the solid κ^*s*^ is used for the compressibility of the artificial fluid. The continuity equation in the artificial fluid domain (Ω̅) can be eventually written as follows,

(30) $$\frac{1}{\kappa^{s}}\frac{\partial p^{f}}{\partial t}+v_{i,i}^{f}=0\text{in}\bar{\Omega }.$$

Using the same semi-implicit interaction force definition and the indicator function as mentioned in the semi-implicit IFEM algorithm, the continuity and momentum equations of the fluid domain, which combines the real fluid domain and artificial fluid domain, can be written as follows,

(31) $$\frac{1}{\kappa^{s}}\frac{\partial p^{f}}{\partial t}I(x)+v_{i,i}^{f}=0\text{in}\Omega .$$

(32) $$\bar{\rho }\frac{\partial v_{i}^{f}}{\partial t}+\bar{\rho }v_{j}^{f}v_{i,j}^{f}=\sigma_{ij,j}^{f}+f_{i}^{FSI,f}\text{in}\Omega .$$

To enforce the assumption ***v**^f^* = ***v**^s^* in Ω̅, a correction force is introduced and added into the fluid-structure interaction force. The correction force, ***f***^Δ***v***^ is defined as,

(33) $$f^{\Delta v}=\rho^{s}(\frac{Dv^{s}}{Dt}-\frac{Dv^{f}}{Dt})\text{in}\Omega^{s}.$$

The correction force is effectively the difference between the material derivative of velocity in the solid and the artificial fluid so that both the inertial and convective acceleration forces are accounted for. It would be zero if the artificial fluid follows the solid exactly. Including this correction force the fluid structure interaction force is redefined as,

(34) $$f^{FSI,s}=\nabla \cdot \sigma^{s}-\nabla \cdot \sigma +f^{\Delta v}\text{in}\Omega^{s}.$$

### 5.2. Outline of the Modified IFEM Algorithm

The algorithm of the modified IFEM is outlined as the following:

1. Solve the solid governing equation Equation (24) with the boundary conditions interpolated from the fluid field in the previous time step,
2. Evaluate the nodal semi-implicit interaction forces ***f**^FSI,s^* on the solid material points, using Equation (34),
3. Distribute the material nodal force onto the fluid grid, from ***f**^FSI,s^* to ***f**^FSI,f^* using interpolation function, Equation (2),
4. Obtain the indicator field by solving Equation (21),
5. Solve for fluid velocities ***v**^f^* and pressure *p^f^* implicitly using Equations (31) and (32),
6. Interpolate the interface velocities and stress from the fluid domain to the material points, Equations (26) and (27), go back to step (1).

## 6. EXAMPLES

In this paper, three biomedical applications are demonstrated. The first example is a blood cell traveling through a bifurcated blood vessel; the second example is to simulate the deployment of an angioplasty stent, which was first presented in [31]; the third example is to study the vocal folds vibration. The first two examples used the original IFEM algorithm where the fluid is blood and the solid is soft tissues. We used the mIFEM algorithm for the third example where the density ratio between the fluid (air) and the soft tissue is large.

### 6.1. Red Blood Cells in a Bifurcated Vessel

Understanding the behavior of red blood cell (RBC) flowing in blood vessels, especially when bifurcation happens, is important in estimating the nonuniform hematocrit distribution that would affect the microvasular oxygen distribution, the effective viscosity of blood in microvessels and the distribution of other metabolites. Learning the behaviors of diseased RBCs that have abnormal rigidity, radius and shape, can be helpful in designing medical therapy. Using the established IFEM method, we can simulate the motion and the deformation of the red blood cell within the vessels, and study in detail how the geometry of bifurcation and fluid field affect and direct which daughter branch the RBC flows.

**Figure 2:**
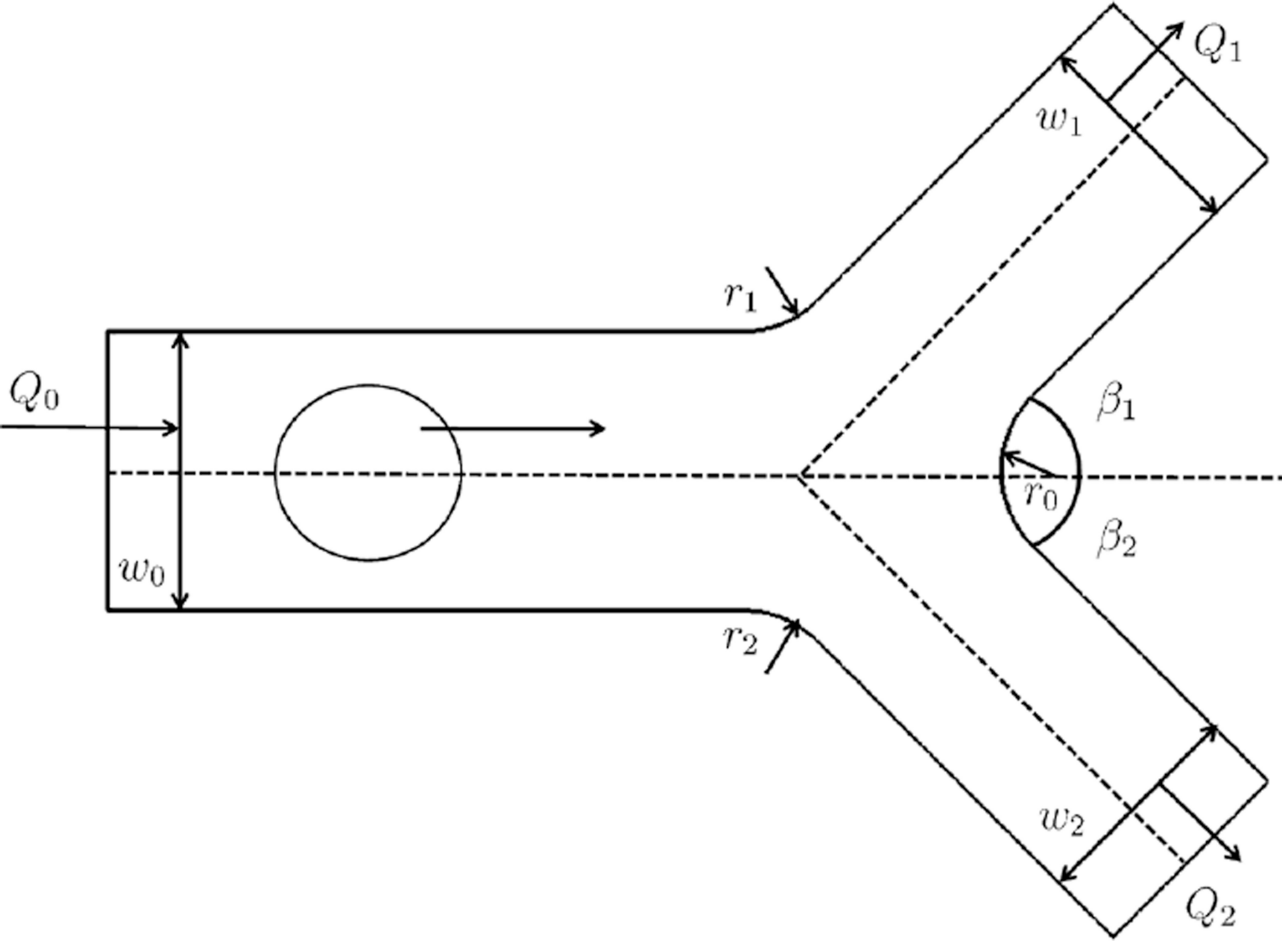
Bifurcation geometry.

The geometry of a bifurcated blood vessel is shown in Figure 2, where *w*_0_ is the diameter of the mother vessel; *w*_1_ and *w*_2_ are the diameters of the daughter vessels on the top and the bottom, respectively; β_1_ and β_2_ are the respective branching angles of the daughter vessels; the branching fillet radii *r*_0_, *r*_1_ and *r*_2_ are given as 3µm to make the vessel branching transition smooth; *Q*_0_, *Q*_1_ and *Q*_2_ represent the flow rate of each vessel. A RBC is placed near the inlet of the vessel. The radius of the RBC is also given as 2.66 µm. The incoming velocity of the mother vessel is set to be a constant as 0.1 cm/s. The branching angles β_1_ and β_2_ are set to be equal and constant, β_1_ = β_2_ = π/4. In this study, we set the diameter of the mother branch to be *w*_0_ = 8 µm, and consider two sets of diameter ratios, *r_d_*= *w*_1_/*w*_2_, to be 1 and 1.44. When the diameter ratio is 1, it is considered as symmetric bifurcation; when it is not 1, then it is considered as asymmetric.

**Figure 3:**
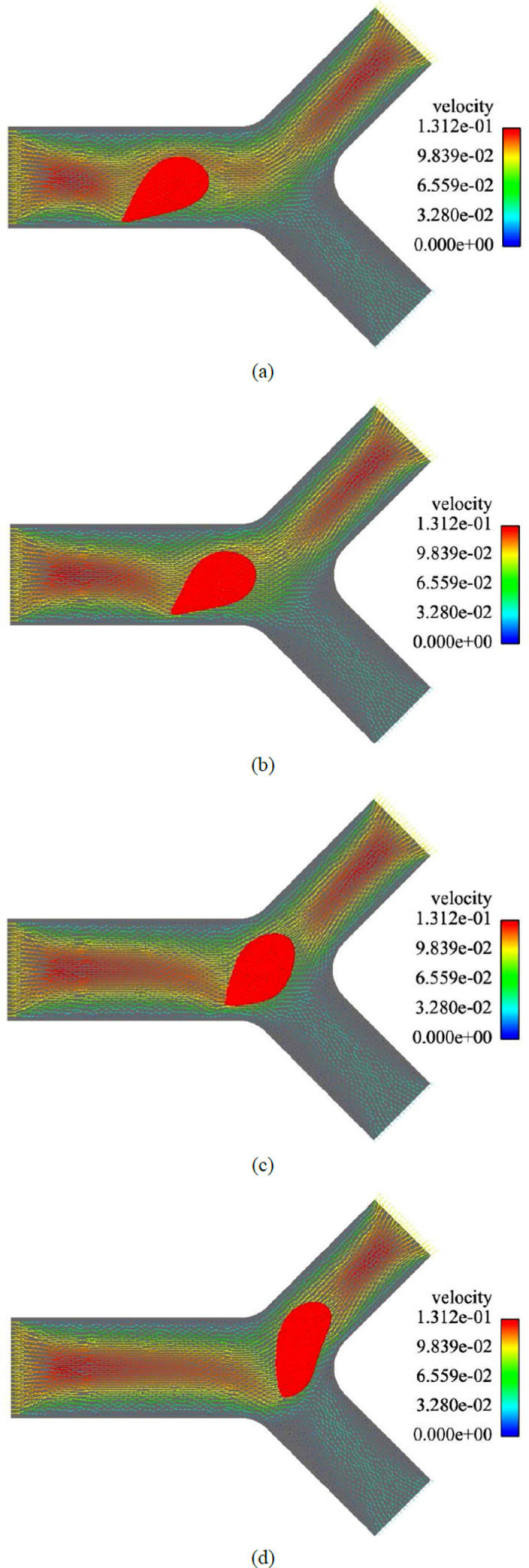
A RBC owing in a symmetric bifurcated vessel with *Q*_1_/*Q*_2_ = 3. (a) *t* = 0.008 s; (b) *t* = 0.012 s; (c) *t* =0.016 s; (d) *t* = 0.020 s.

**Figure 4:**
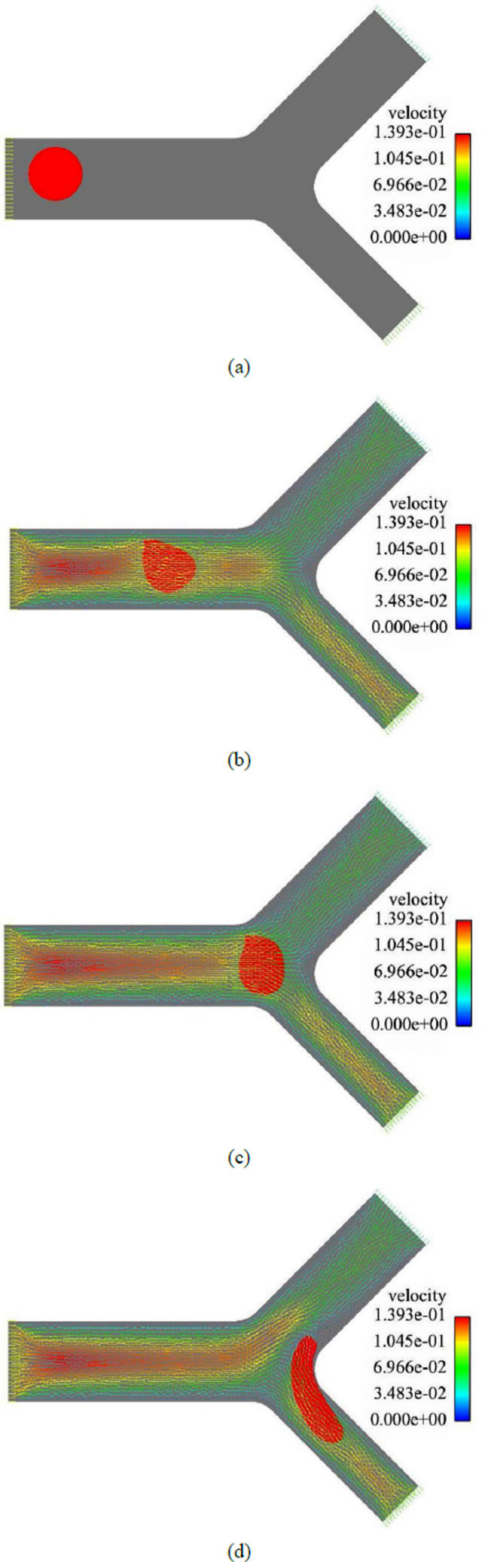
A RBC flowing in an asymmetric bifurcation vessel with *Q*_1_/*Q*_2_ =1. (a) *t* = 0.00 s; (b) *t* = 0.01 s; (c) *t* =0.02 s; (d) *t* = 0.03 s.

Figures 3 and 4 represent the blood cell behaviors when encountering bifurcation in symmetric and asymmetric vessels with different original positions and ratios of flow rate of each daughter vessel. Based on Figure 3 we can notice that although the daughter branches are symmetric in the geometry, due to the asymmetric boundary conditions where the ratio of the flow rates for the two daughter branches is *Q*_1_/*Q*_2_ = 3, the blood cell tends to move to the daughter branch with a higher mass flow rate. When the daughter branches are asymmetric in geometry but with the same mass flow rate *Q*_1_/*Q*_2_ = 1, as shown in Figure 4, the blood cell moves to the one with a smaller cross section area, which is due to the higher average velocity in that daughter branch.

**Figure 5:**
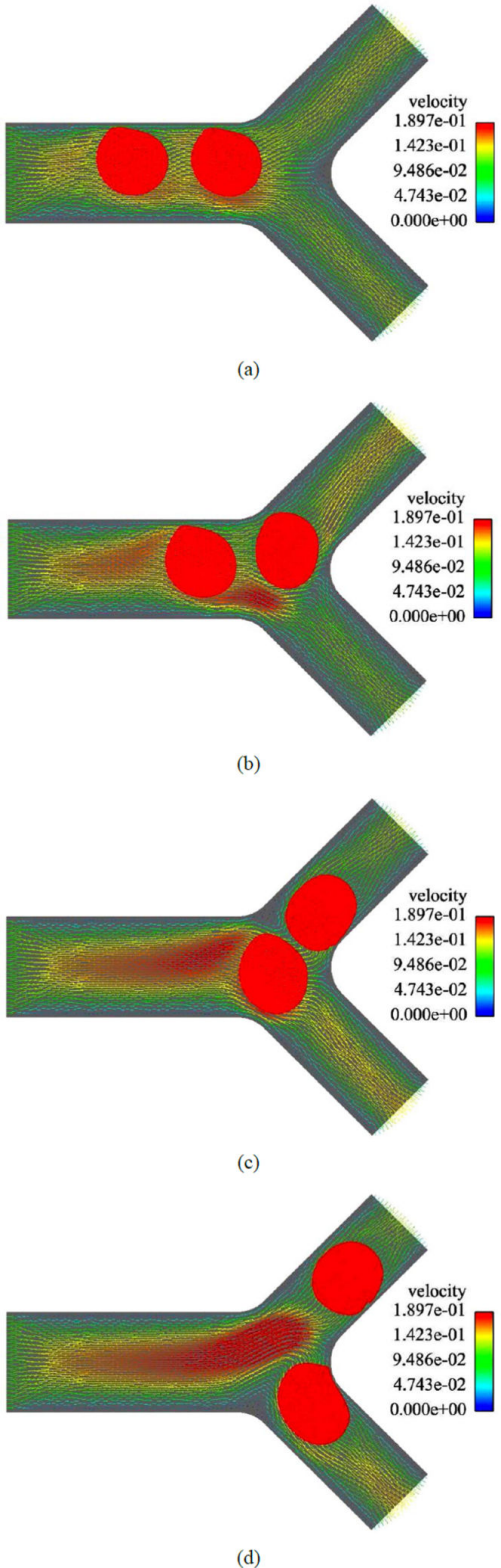
Two RBCs in a symmetric bifurcation. (a) *t* = 0.004 s; (b) *t* = 0.008 s; (c) *t* =0.012 s; (d) *t* = 0.018 s.

A multi-blood cell case is also simulated and the result is shown in Figure 5. The cell-to-cell interaction can be thought as two parts: 1) one cell changes the deformation and motion of the other cells by directly contacting each other; 2) one cell affects the other cells indirectly by changing the surrounding fluid field. For this case, instead of specifying the flow rate ratio between the daughter vessels, a constant inlet flow rate is given. Therefore, by simulating two RBCs laying in a line profile going through the symmetric bifurcation together, we are able to see this indirect cell-to-cell interaction. From these simulation results, the IFEM is proved to be suitable to simulate the red blood cell motion and deformation in microvessel bifurcation.

### 6.2. Deployment of Angioplasty Stents

For individuals with an occlusive vascular disease, blood flowing to an organ or to a distal body part is impaired by narrowed arteries with fatty deposits or calcium accumulations. Angioplasty was introduced by Dr. Andreas Gruentzig in the mid to late 1970’s and is widely used today. The area of arterial blockage is dilated with the help of a catheter that has an inflatable small balloon at its tip. Then, the plaque is squeezed along the artery wall. A decade later, stenting was introduced by Dr. Julio Palmaz in 1988 to improve the angioplasty procedure. Like angioplasty, coronary stents physically open the channel of constricted arterial segments. During stenting, a catheter delivers a balloon and a surrounding stent to the location of the blockage area. The balloon deploys the stent, remains inflated for 30 seconds and then is deflated. At the end of the process, the expanded stent is embedded into the wall of the diseased artery and holds it open.

Here, we mainly focus on the deployment process of balloon-expanding stents. Balloon expandable stents are typically made of stainless steel tubing mounted over an angioplasty balloon and then plastically deformed to their final diameter by high pressure balloon inflation.

Colombo *et al*. [39] and Goldberg *et al*. [40] demonstrated that stent apposition was inappropriate in up to 87% of the cases using conventional balloon implantation techniques. Therefore, researchers started to study the role of stent deployment in order to define an optimal deployment technique. Deviations from normal blood flow pattern may favor the initiation and progression of a vascular wall lesion. These conditions are certainly fulfilled when a stent is deployed in the vessel wall, completely or incompletely. Segers *et al*. [41] showed experimentally the importance of optimal stent deployment for steady state condition and pulsatile flow by studying the most important stents parameters that influence hemodynamics. Russo *et al*. [42] suggested that the use of high pressure non-compliant balloon stent deployment techniques give better initial results. However, these stents provoke an increased intimal growth due to more profound and deep vascular injury. Shear stress, exerted on both the vascular wall and on the blood constituents, is considered to play an important role in restenosis. Therefore, besides biocompatibility issues, the design of both the stent and delivery mechanisms should receive the necessary attention during the development process of the future generation of stents.

**Figure 6:**
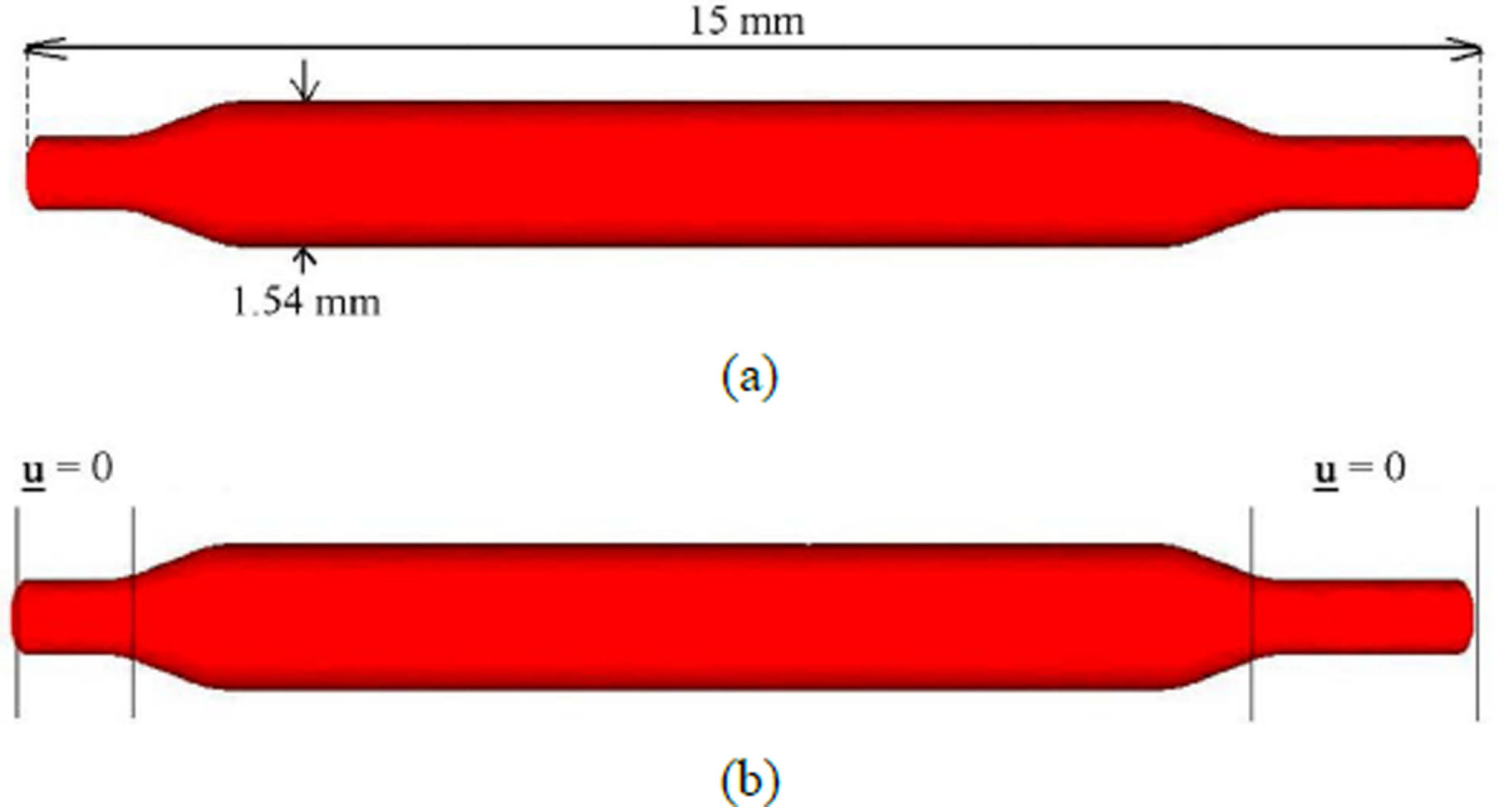
Balloon geometry setup. (a) Initial balloon geometry before inflation; (b) Fixed boundaries applied at the two ends of the balloon.

In our previous work [27], we built a computer model and simulated a balloon expandable stent interacting with its surrounding fluid using the immersed finite element method. The initial configuration of the balloon and stent are designed and discretized using SolidWorks [43] and imported into our IFEM code. With the previously derived computational algorithm, the displacement and stress distribution of the stent and the velocity profile of the fluid domain can be obtained and analyzed. A balloon is designed with deflated tips at its two ends in its initial undeformed configuration, as shown in Figure 6 (a). A catheter is located inside the balloon to apply appropriate pressure to inflate the balloon. The balloon has a length of 15 mm, an outer diameter of 1.54 mm, and a thickness of 0.04 mm. Both ends of the balloon are fixed in all directions as shown in Figure 6(b).

The balloons used for stenting are made of very stiff polyamide (nylon) material [44,45]. The balloon is modeled as hyperelastic material with Mooney-Rivlin description in the simulation. The parameters and material properties used for the balloon are summarized in Table 1.

**Figure 7:**
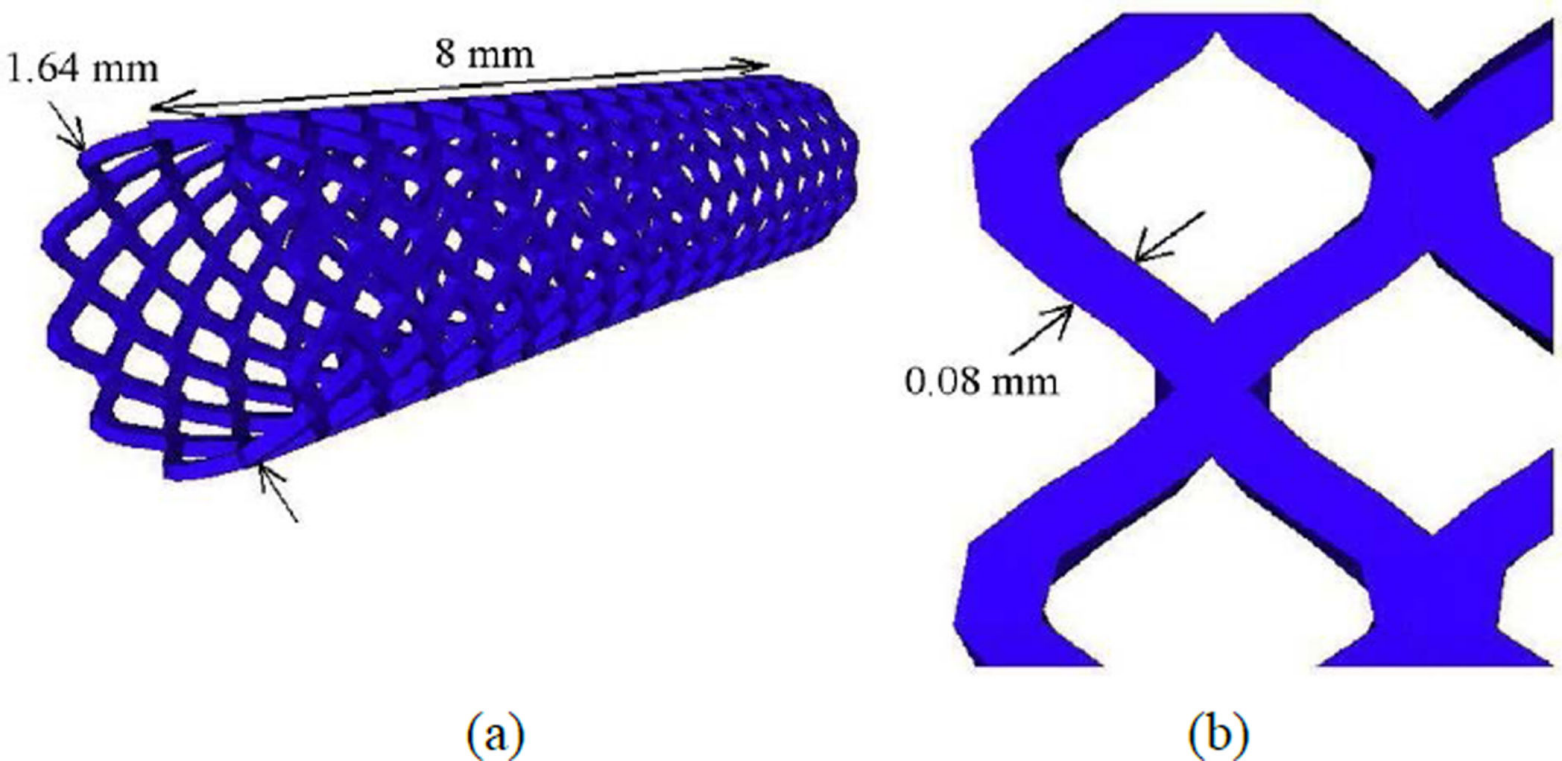
Design of a Medtronic AVE Modular stents S7.

A stent is a cylindrical and symmetrical assembly of inter-connected diamond-shaped members. In this particular model, we use the Medtronic AVE Modular stents S7 (Medtronic AVE, Inc., Santa Rosa, CA, USA), Figure 7(a). Although this stent is no longer been widely used, it is still a good representation of a typical geometrical shape of a stent, the model can be simply modified to other expandable devices. The stent, made of wires that form a diamond shape as shown on Figure 7(b), has an outer diameter of 1.64 mm. The stent is made of 16 identical structural members with a total length of 8 mm before its expansion. The cross-section of the wire has a width of 0.08 mm. The structural members are connected peak-to-peak at their tips. Balloon-expandable stents are made from materials that can be plastically deformed through the inflation of a balloon. An ideal stent should have low yield stress (to make it deformable at manageable balloon pressures), high elastic modulus (for minimal recoil), and high strength through expansion. The most widely used material for this type of stents is stainless steel, typically 316 L, a particular corrosion-resistant material. For stents the fatigue resistance is of high importance. Therefore, the microstructure requires very small grain sizes. The typical average grain sizes of approximately 25 µm exist in stainless steel stents and 8 to 9 grains over the strut wall thickness are desired [46]. We assume a clean homogeneous material with a uniformly fine grain size and a near 100% density. Stainless-steel alloys are usually preferred to design stents because they have been proven to be biocompatible for long-term implants in the human body and easily deformable in fully annealed condition. The stent is discretized using 9.979 nodes and 16.888 elements. A summary of the properties and parameters used for the stent is listed in Table 2.

**Figure 8:**
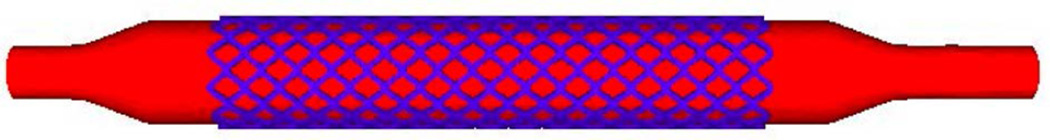
Initial configuration of catheter, ballon, and stent.

Finally, the stent is mounted around the balloon as shown in Figure 8. The stent is placed at the center of the balloon. At this point, several assumptions must be made: 1) the force applied on the balloon is transferred directly to the stent; 2) there is no fluid between the balloon and the stent; and 3) the contact surface between the balloon and the stent is frictionless.

The fluid domain represents blood in the artery where the stent is deployed. We consider a straight artery segment with a length of 16 mm and a lumen diameter of 4.5 mm. Our computational model assumes that the fluid is everywhere in the domain. Although the balloon is not inflated with the same fluid as the surrounding blood, a liquid (with color) that has similar properties as the blood is normally inserted into the balloon for easier visualization in surgeries. Therefore, it is a reasonable assumption to have homogeneous fluid in the entire computational domain. Blood is an incompressible fluid consisting of a suspension of deformable particles (blood cells, platelets, etc.) in a Newtonian liquid (plasma). Except within the microcirculation, blood can be considered homogeneous with constant density and viscosity. The parameters and properties used for the fluid domain to perform the simulation can be found in Table 3.

**Figure 9:**
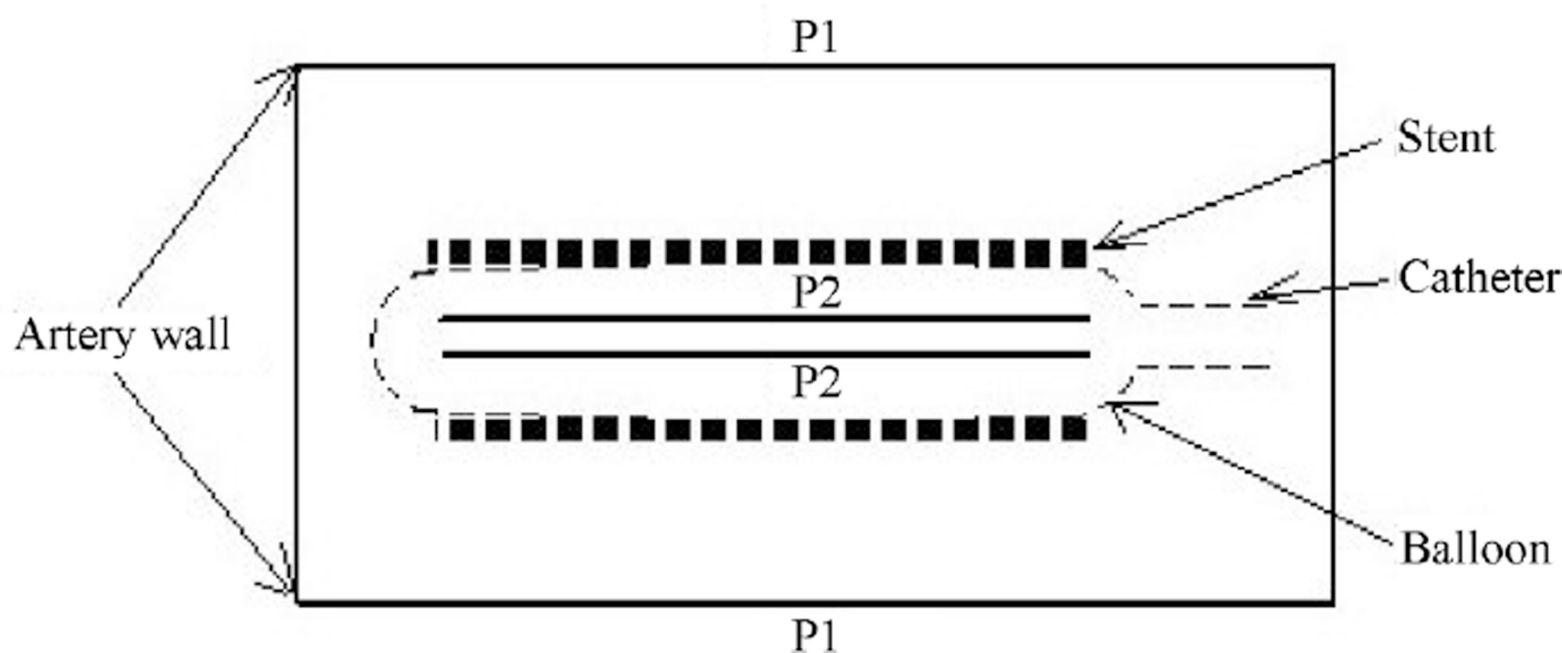
The initial conditions applied to the fluid domain: constant pressure difference (*P*_2_ − *P*_1_) is applied from the catheter to the fluid boundaries.

The crimped stent is deformed by a radial force from the balloon. Experiments performed by Dumoulin and Cochelin [47], and Moore and Berry [48] showed that, except at the tips, the structure is almost uniformly dilated and finally expanded. Thus, it seems justifiable to model expansion by considering a long structure under uniform radial internal pressure. This pressure difference is applied from the centerline towards the outer diameter of the balloon and the artery wall, as shown on Figure 9. The applied inflation pressure is dependent on the struts of the wires and the specific material used. The total inflation pressure for the balloon is equal to the sum of the pressure required to deform the balloon material and to deploy the stent. During the entire simulation the inflation pressure is constant and equals to 100 g/cm^2^.

**Figure 10:**
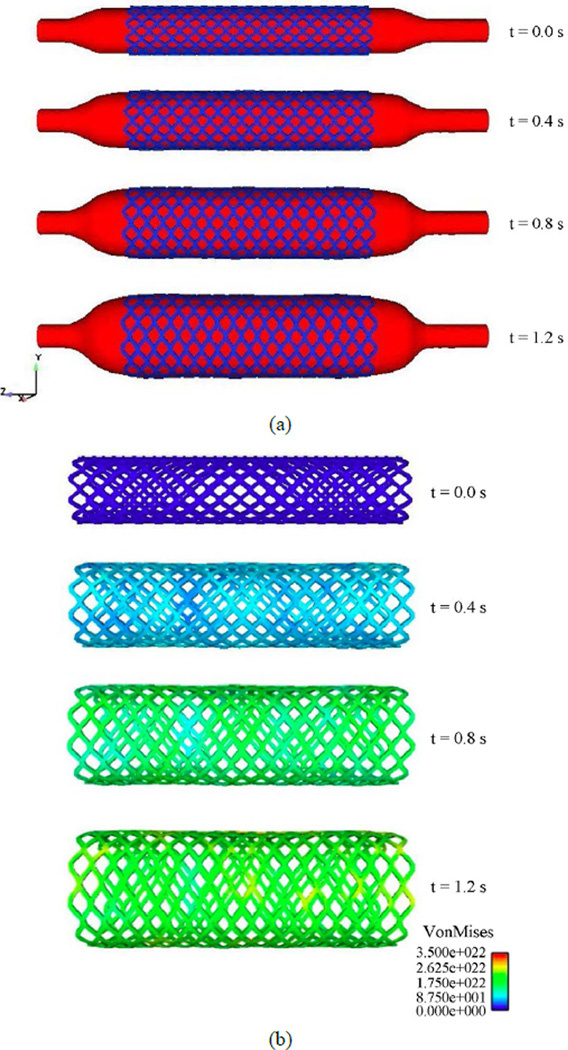
Stent configuration and von Mises stress map during stent deployment. (a) Stent deployment process; (b) Von Mises stress during stent deployment.

Our primary goal is to study the deformation of the stent during its deployment. Figure 10(a) shows the deployment of the stent during balloon expansion at different time steps. The pressure applied onto the fluid inflates the balloon and the balloon provides a force onto the stent, which enables the stent to expand radially outward until it contacts the inner surface of the artery wall.

**Figure 11:**
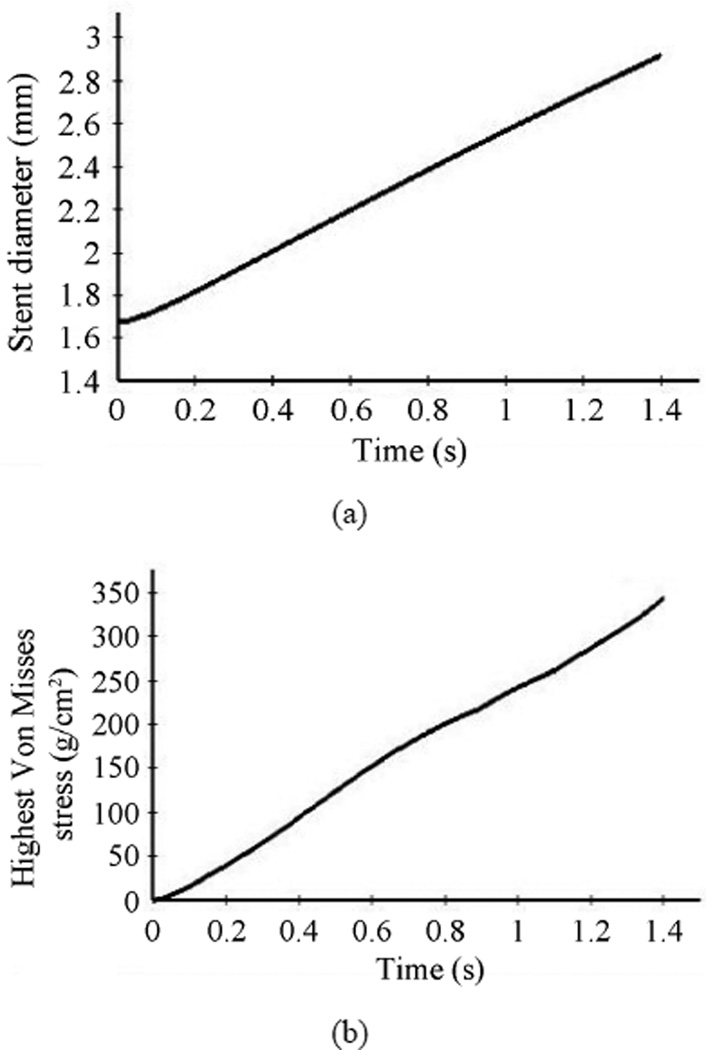
Stent diameter and maximum von Mises stress vs. time during stent deployment. (a) Stent diameter variation during the deployment; (b) Maximum Von Mises stress variation during the deployment.

During deployment, the diameter of the stent increases from 1.64 mm to 2.82 mm. As expected, the stent deforms uniformly in its radial direction, expanding by 1.7 times its initial diameter for our given material. Figure 11(a) shows that the expansion of the stent in its radial direction follows a linear variation.

Figures 10(a) demonstrates how the diamond shape of the stent deforms during the deployment. Compared to experiments and observations of deployment of a real stent, our three-dimensional computational model gives a realistic representation of stent expansion during balloon inflation.

**Figure 12:**
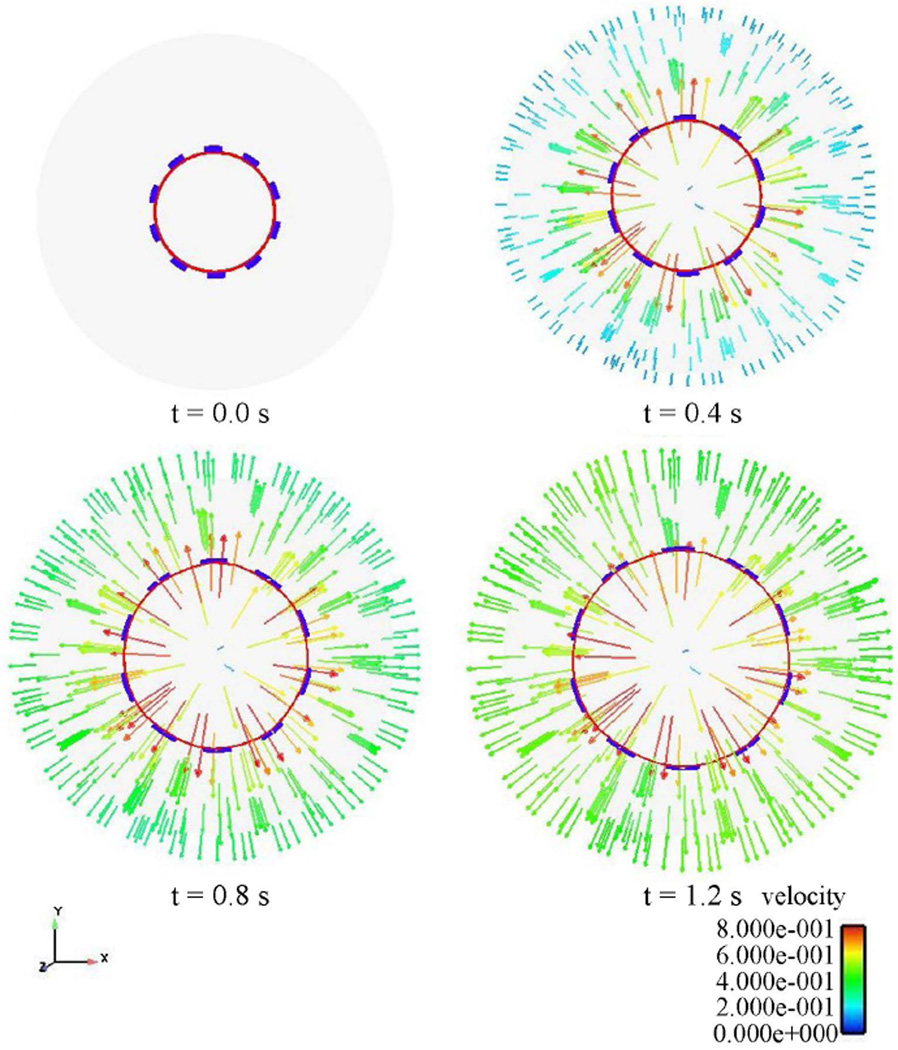
Radial fluid velocity profile at different time steps.

**Figure 13:**
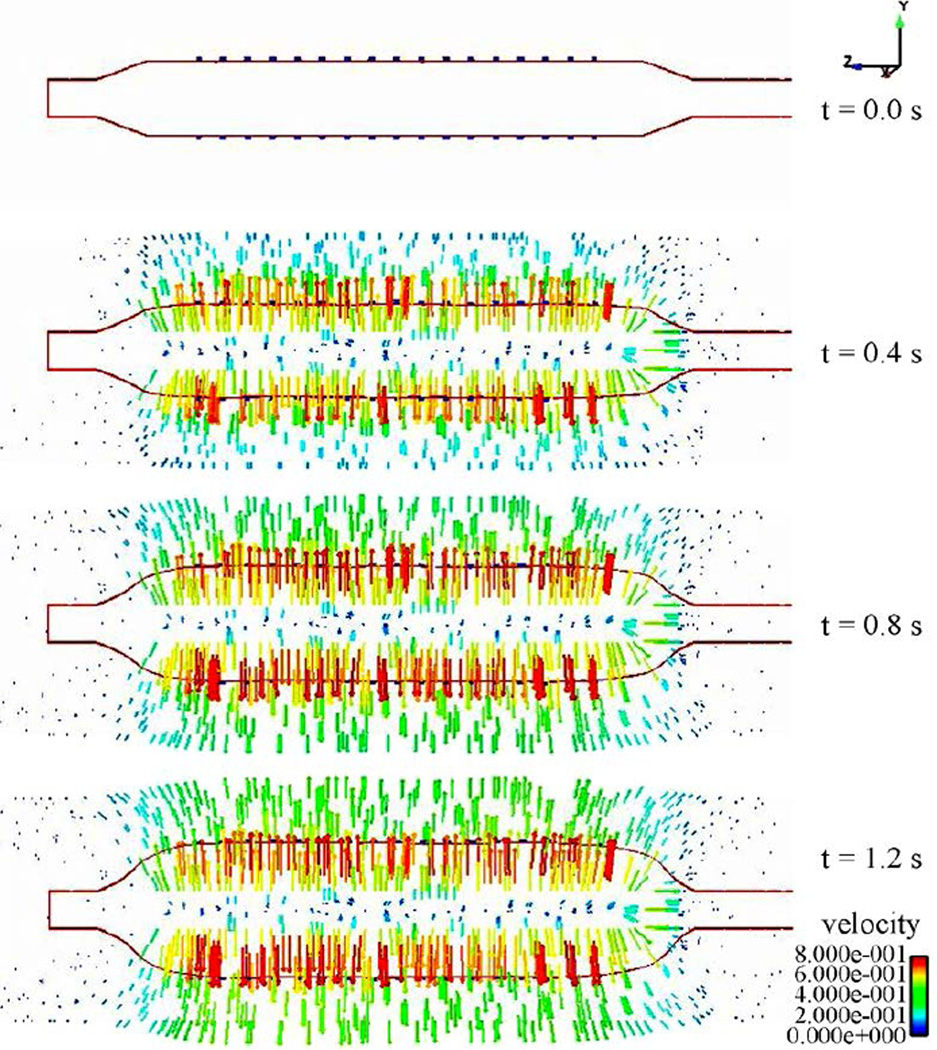
Velocity profile in the longitudinal direction during expansion at different time steps.

The strength and the long term in-vivo performance of the stent can be determined from the stress distributions with the goal of minimizing vascular injury. The Von Mises stress distribution along the stent is reported at different time steps in Figure 10(b). The stress distribution is uniform longitudinally along the stent and varies during deployment. The highest stress values appear at the final stage of expansion, and the peak Von Misses stress follows a linear variation during the entire simulation as shown on these values are critical for recoil and failure analysis. It can be concluded that the stress distribution is a function of applied pressure, balloon and stent material properties, fluid properties, and stent geometry. Figure 12 shows the radial fluid velocity profile during the deployment of the stent. This profile represents better how the applied uniform pressure acts on the balloon in the radial direction. Figure 13 shows the magnitude and profile of the velocity in the transverse plane. Both figures demonstrate a uniform velocity distribution in the direction of expansion, illustrating how well our model simulates the expansion mechanism through applied pressure difference.

### 6.3. Human Vocal Folds Vibration during Phonation

**Figure 14:**
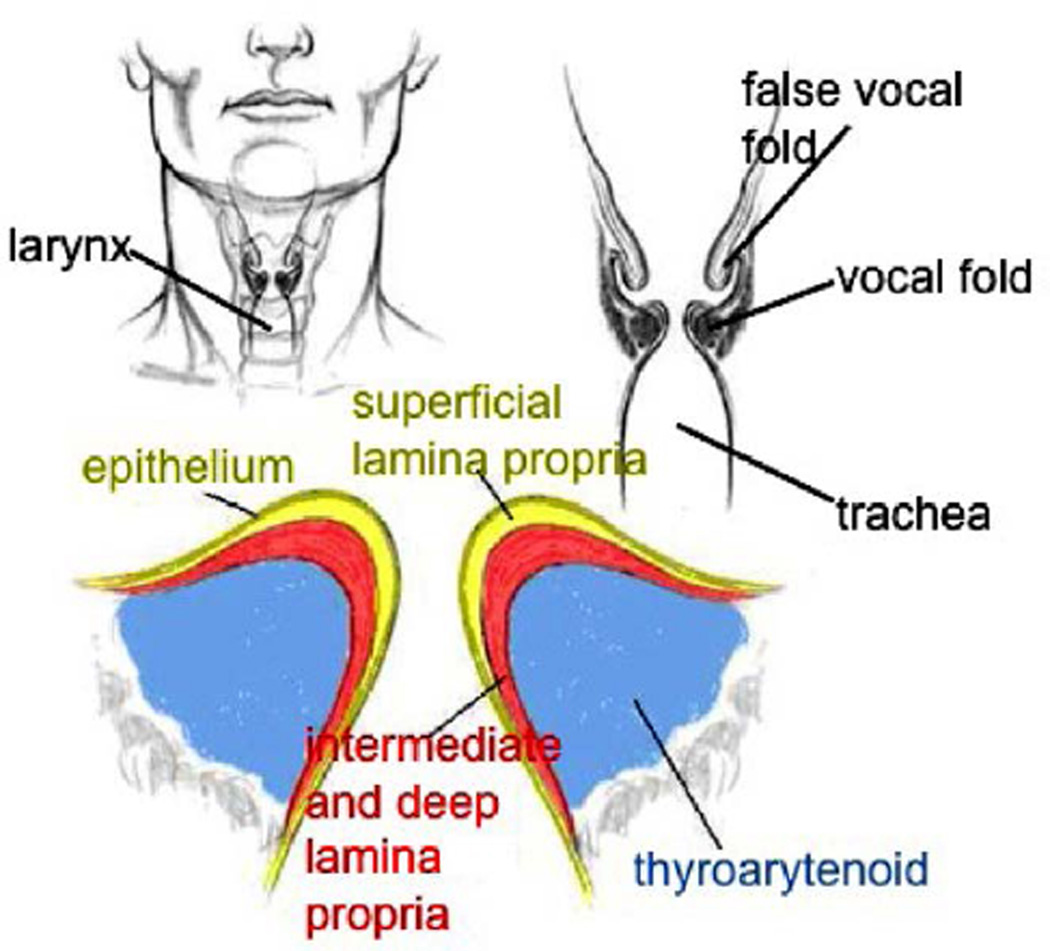
Human vocal folds.

Voice is produced by the vibration of vocal folds. The vocal folds are a pair of pliable structures located within the larynx at the top of the trachea, see Figure 14. The human vocal folds are roughly 10 – 15 mm in length and 3 – 5 mm thick. The human vocal folds are laminated structures composed of five different layers: the epithelium, the superficial layer (SLP), the intermediate layer (ILP), the deep layer and thyroarytenoid muscle, shown in Figure 14.

An accurate numerical simulation of the vocal folds vibration can help us obtain a better understanding of the dynamics of the voice production in human beings. Due to the complicated nature of this problem, the numerical model has to fulfill the following requirements. First, the numerical model has to represent completely a coupled fluid-structure interaction system. Second, the numerical model should perform well when there exists large density ratio between the fluid and structure because the density of the vocal fold muscle is close to water and the density ratio between the vocal fold muscle and the airflow is about 1000. Third, the motion and deformation of the structure have to be predicted accurately with complicated geometry and material descriptions because the vocal folds have complex shape and layer-structures and are viscoelastic materials. The mIFEM is a perfect numerical method to perform the simulation of this complex problem.

**Figure 15:**
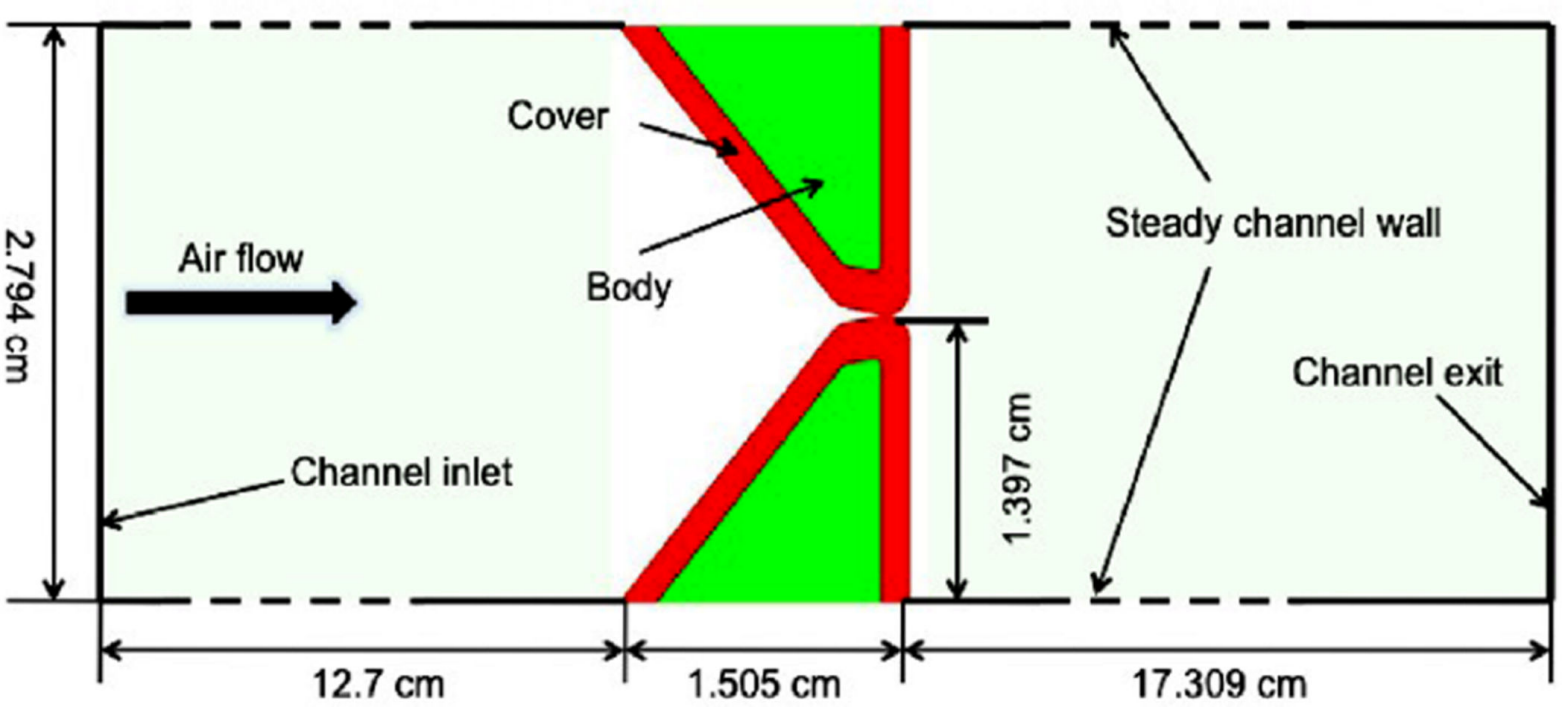
2-D two-layer self-oscillated vocal folds model.

The geometry of the self-oscillated vocal folds model is shown in Figure 15. Since sound is generated by the compression of air, the working fluid is taken as compressible air governed by the ideal gas law at room temperature. The density of the fluid is

ρ^*f*^ = 1.3×10^−3^g/cm^3^ and the viscosity of the fluid is
μ = 1.8×10^−4^ g/cm·s.

The vocal fold muscle is considered as isotropic viscoelastic material. The vocal fold is assumed to have layered structure, outside cover layer (red) and inside body layer (green). The cover layer is much softer than the body layer. For the cover layer the Young’s modulus is *E* = 10 kPa, whereas *E* = 40 kPa for the body layer. The densities of both cover and body layer are assumed to be the same as ρ^*s*^ = 1.0g/cm^3^. The Poisson ratio is ν = 0.3. Two vocal folds have the exact same geometry and material description, sit in the fluid channel symmetric about the central line. A constant total pressure boundary condition of *P_in_* = 1 kPa is applied at the channel inlet and the outflow boundary is given at the channel exit. No-slip and no-penetration boundary conditions are applied on the channel walls and on the vocal fold surfaces.

**Figure 16:**
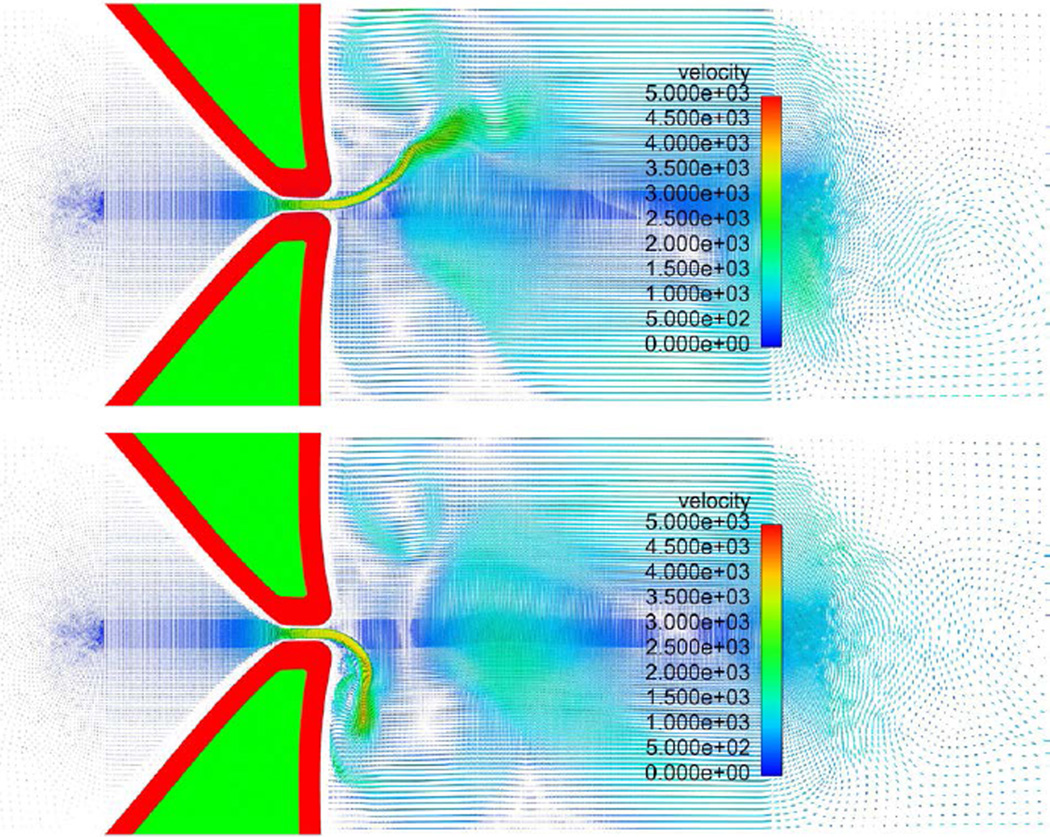
Fluid velocity field at two typical instances during steady vibration.

A snapshot of the fluid velocity field at two typical instances during a steady vibration cycle are shown in Figure 16. One can see that the fluid field is not symmetric about the central line during the vibration. The glottal jet tends to attach to one side of the vocal folds randomly, which is the so-called the “Coanda effect” [49,50].

**Figure 17:**
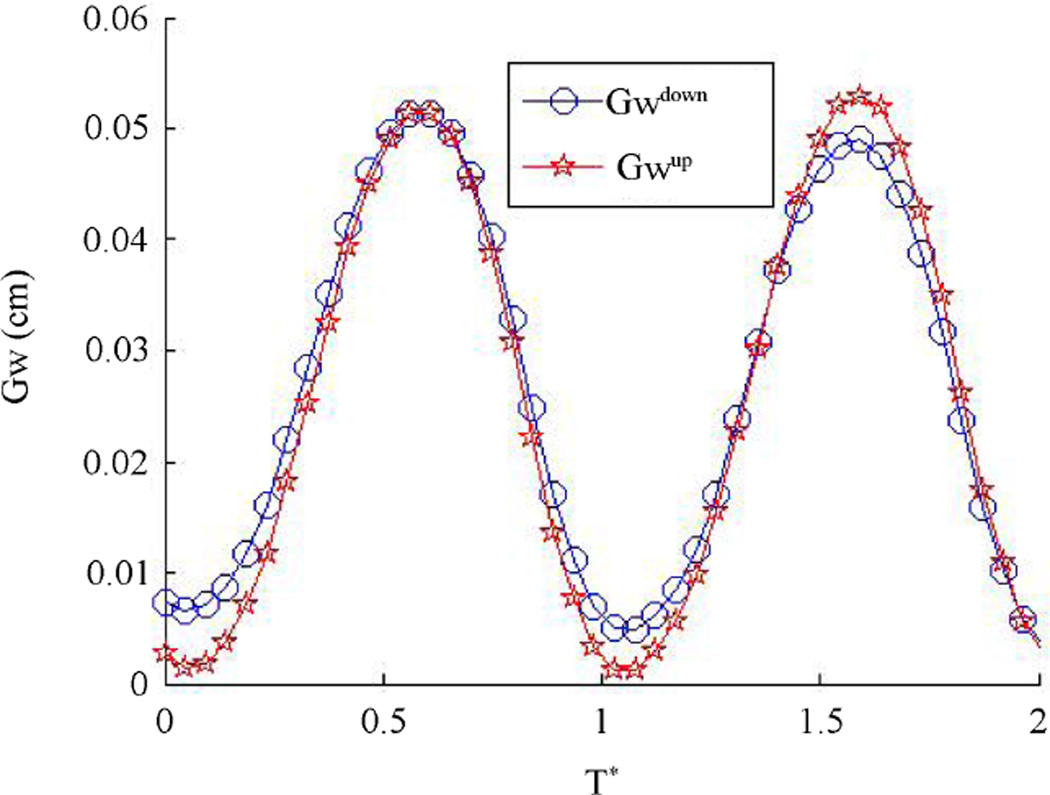
Half glottis width of top and bottom vocal folds.

**Figure 18:**
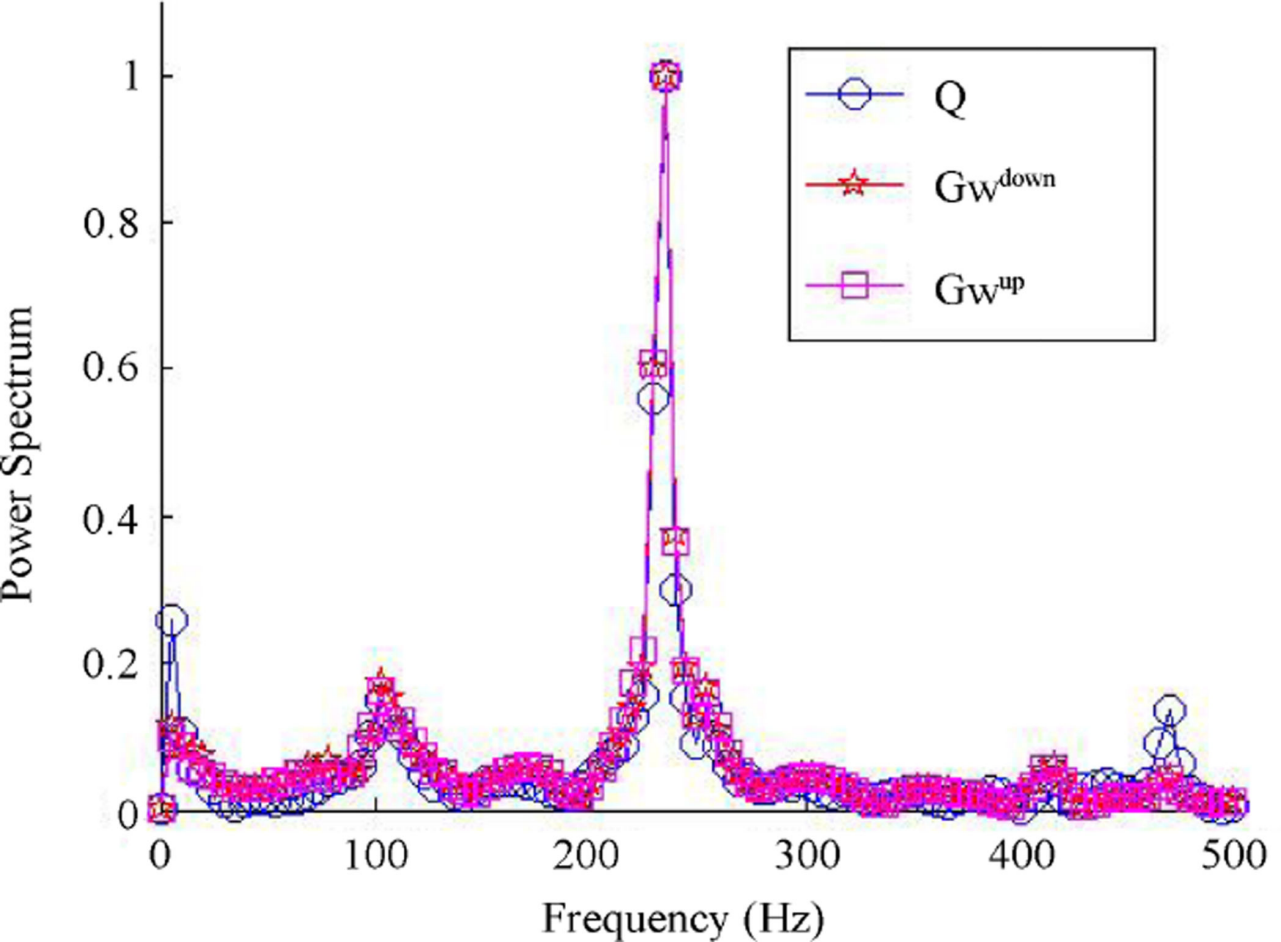
Spectrum plot of flow rate and half glottis width of the top and bottom vocal folds.

The asymmetrical airflow causes an asymmetrical pressure distribution in regions near the vocal folds and change in the vibration pattern. The minimum distance between the vocal fold surface and the central line is measured to represent the half glottis width (*Gw*), shown in Figure 17, where *Gw_up_* and *Gw_down_* represent the opening width for the up and down vocal folds, respectively. This figure shows that the simulation captures the vocal folds to have a repeated opening and closing process. When the glottis width is zero or near-zero, then the vocal folds are closed, there is no air flowing through. The pressure starts to build up in the upstream of the vocal channel. As the pressure increases, it starts to push the vocal folds to open and eventually reaches a maximum glottis width, the high pressure is released. The vocal folds then return back to its closed position, and the whole process restarts. The glottis width for the up and down vocal folds do not equal each other over cycles, indicating that the vocal folds motion is asymmetric although the vibrational frequency is found to be the same. The vibrational magnitude is slightly off from each other. To find out the vibration frequency, FFT is performed on the up and down glottis widths and the volume flow rate, *Q*. The power spectra are plotted in Figure 18 where the frequency for these three variables are the same and found to be 234 Hz, which is in the expected range of a female vocal fold vibrational frequency.

## 7. CONCLUSION

In this paper, we reviewed IFEM algorithms that had been developed over the past decade. The IFEM method is a numerical scheme that adopts the non-boundary-fitted mesh approach and fully couples the fluid-structure interaction by interpolation of the interacting domains. The fluid and solid domains are solved independently using finite element method and coupled with each other within one time step through fluid-structure interaction force. The original IFEM algorithm is considered as explicitly coupling for the fluid and solid, which can lead to instability issue when time step is not sufficiently small. The semi-implicit IFEM algorithm tackles this problem by modifying the FSI force and adopting the concept of the indicator function. It extends the stability range of the numerical scheme and allows us to consider the fluid-structure interaction problems when the fluid and solid properties are very different from each other, for example high density ratio between the solid and fluid, and the solid with relatively large stiffness. For high Reynolds number flows,, the modified IFEM algorithm performs better due to its accurate description of the solid motion and deformation through capturing the dynamics of the solid motion. Three biomedical applications are studied using these IFEM algorithms to simulate soft tissues interacting with fluid where the fluid can be either blood or air.

## Figures and Tables

**Table 1:** Parameters and material properties used for the balloon.

5929 nodes	Length = 15 mm	Density= 3000 kg/m^3^
17,846 elements	Diameter = 1.54 mm	Shear modulus = 300 Pa
	Thickness = 0.04 mm	Bulk modulus = 1500 Pa

**Table 2:** Parameters and material properties used for the stent.

9979 nodes	Length = 8 mm	Density = 4000 kg/m^3^
16,888 elements	Diameter = 1.64 mm	Shear modulus = 300 Pa
	Thickness = 0.08 mm	Bulk modulus = 1500 Pa

**Table 3:** Parameters and properties used for the fluid domain.

4348 nodes	Length = 16 mm	Density = 1000 kg/m^3^
20,568 elements	Diameter = 4.5 mm	Viscosity = 0.01 N·s/m^2^
